# Wavelength-Dependent Effects of Photobiomodulation for Wound Care in Diabetic Wounds

**DOI:** 10.3390/ijms24065895

**Published:** 2023-03-20

**Authors:** Peter Dungel, Sanja Sutalo, Cyrill Slezak, Claudia Keibl, Barbara Schädl, Harald Schnidar, Magdalena Metzger, Barbara Meixner, Jaana Hartmann, Johannes Oesterreicher, Heinz Redl, Paul Slezak

**Affiliations:** 1 Ludwig Boltzmann Institute for Traumatology, The Research Center in Cooperation with AUVA, 1210 Vienna, Austria; sanja.sutalo@fu-berlin.de (S.S.); cslezak@uvu.edu (C.S.); claudia.keibl@jku.at (C.K.); barbara.schaedl@trauma.lbg.ac.at (B.S.); magdalena.metzger@trauma.lbg.ac.at (M.M.); barbara.meixner@rockfishbio.com (B.M.); jaana.hartmann@trauma.lbg.ac.at (J.H.); johannes.oesterreicher@trauma.lbg.ac.at (J.O.); heinz.redl@trauma.lbg.ac.at (H.R.); paul.slezak@trauma.lbg.ac.at (P.S.); 2 Austrian Cluster for Tissue Regeneration, 1200 Vienna, Austria; 3 Department of Physics, Utah Valley University, Orem, UT 84058, USA; 4 University Clinic of Dentistry, Medical University of Vienna, 1090 Vienna, Austria; 5 SCARLETRED Holding GmbH, 1030 Vienna, Austria; harald.schnidar@scarletred.com

**Keywords:** photobiomodulation, wound healing, light emitting diodes, diabetes

## Abstract

Photobiomodulation, showing positive effects on wound healing processes, has been performed mainly with lasers in the red/infrared spectrum. Light of shorter wavelengths can significantly influence biological systems. This study aimed to evaluate and compare the therapeutic effects of pulsed LED light of different wavelengths on wound healing in a diabetic (db/db) mouse excision wound model. LED therapy by Repuls was applied at either 470 nm (blue), 540 nm (green) or 635 nm (red), at 40 mW/cm^2^ each. Wound size and wound perfusion were assessed and correlated to wound temperature and light absorption in the tissue. Red and trend-wise green light positively stimulated wound healing, while blue light was ineffective. Light absorption was wavelength-dependent and was associated with significantly increased wound perfusion as measured by laser Doppler imaging. Shorter wavelengths ranging from green to blue significantly increased wound surface temperature, while red light, which penetrates deeper into tissue, led to a significant increase in core body temperature. In summary, wound treatment with pulsed red or green light resulted in improved wound healing in diabetic mice. Since impeded wound healing in diabetic patients poses an ever-increasing socio-economic problem, LED therapy may be an effective, easily applied and cost-efficient supportive treatment for diabetic wound therapy.

## 1. Introduction

Incidence of diabetes has risen to an alarming rate. In Europe, about 60 million people are affected from diabetes and each year about 3.4 million people die from the consequences of elevated blood sugar levels [1,2]. Due to the chronic state of hyperglycemia in diabetic patients, an unbalanced level of matrix metalloproteases (MMP) establishes, which leads to excessive degradation of the extracellular matrix, ultimately leading to reduced tensile strength of the skin [3]. Along with other factors, including limited functionality of leukocytes and endothelial cells, as well as decreased collagen deposition by fibroblasts, this results in defective wound healing [4]. Patients diagnosed with diabetes exhibit a 25% lifetime prevalence of developing diabetic foot ulcer (DFU) [5,6]. The chronic impairment of wound healing predisposes affected patients to severe infections, leading to the fact that one out of six DFU patients will require limb amputation, with a following 5-year mortality rate of up to 77% [7]. Those numbers emphasize the huge burden of impaired diabetic wound healing. However, to date, no adequate therapeutic approaches exist. In recent decades, different therapeutic approaches based on biophysical effects to treat diabetes-related impaired wound healing have been investigated. These therapeutic options include extracorporeal shock wave treatment, ultrasound, negative and positive pressure, and photobiomodulation or photobiomodulation by laser or light emitting diode (LED) irradiation, and have been demonstrated to have clinical benefits for patients [8,9,10]. Although the therapeutic benefit of these therapies could be shown, for most approaches, the involved molecular mechanisms have not yet been satisfyingly unraveled. Photobiomodulation through LED irradiation is remarkably interesting and stands out from other biophysical methods as it is cost-effective, easy to apply and safe to use.

A recent study reviewed four randomized control trials involving 131 participants, with the main aim to confirm the beneficial effects of PBM for the treatment of DFU. All studies demonstrated positive healing outcomes by PBM compared to placebo or control groups, and no adverse events associated with PBM treatment were reported [11,12]. Most of the studies on PBM in wound healing to date have been performed on red to near infrared (red-NIR) light sources, ranging from 600–1400 nm. However, shorter wavelengths could support wound healing probably via alternative modes of action. Light can significantly influence biological systems such as nitric oxide (NO) metabolism. We showed previously that photolysis of bioactive NO from nitrosyl-hemoglobin or mitochondrial protein complexes is wavelength-dependent and significantly more efficient with light in the green and blue range [13,14]. Nitric oxide has been shown to be highly involved throughout all phases of wound healing where it is associated with the regulation of inflammatory response, collagen deposition and angiogenesis [15].

In the present study, the effects of low level light therapy of three different wavelengths (red, 629 nm; green, 540 nm; blue, 470 nm) on wound healing in a diabetic (db/db) mouse excision wound model was investigated.

## 2. Results

**Figure 1:**
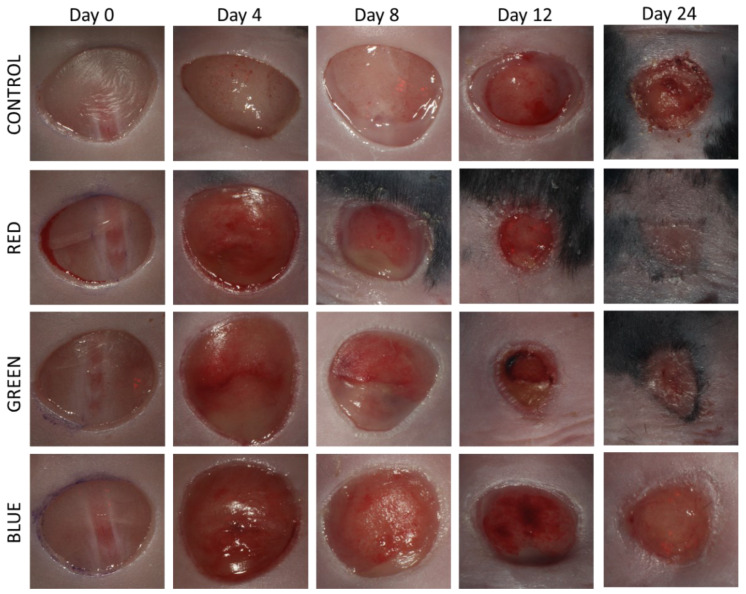
Typical progression of wound healing in untreated control animals and of wounds treated with pulsed LED photobiomodulation with red, green or blue light, respectively.

All used animals in the impaired wound healing group showed blood glucose levels ≥300 mg/dL. Figure 1 shows representative figures of wounds and wound progression over time.

**Figure 2:**
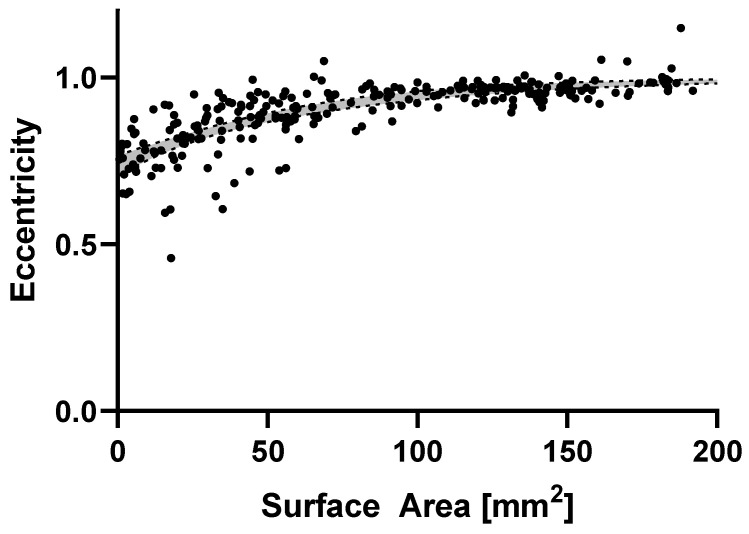
Wound shape eccentricity as measured by the ratio of circular radii estimates determined from area and circumference, respectively, for corresponding wound size (mm^2^). Black dots represent represent these values forall timepoints are shown indicating a well-controlled wound shape of the experimental data as can be seen by the narrow mean prediction bands at the 95% confidence interval (grey) of exponential regression lines.

Wound geometry is an important factor of the healing process and has to be considered explicitly, especially when considering challenges associated with circular excisions [16]. Especially in earlier wound stages, maintaining a circular shape is crucial as to not introduce other driving mechanisms in addition to phototherapy into the system. Figure 2 shows that, for the study group, we maintain a high level of circularity until wounds have almost closed up. We have measured eccentricity as the ratio of the circular estimates for the radii based on the circumference and area. A perfect circle would yield a value of 1 and a linear cut would correspond to a 0. Here, highly non-circular wounds starting in early stages would tend to smaller wound sizes at increasingly elongated wound shapes. This is evident when fitting the data using a least squares exponential plateau regression fit. Within our study group we see a certain geometric homogeneity as evident by the narrow mean prediction bands at the 95% confidence interval. Furthermore, there is no observed difference in the wound shapes for each of the experimental therapy groups as measured by the distribution of eccentricities (F(21, 222) = 0.4744, *p* = 0.9768) as they develop very similar shapes as they approach final healing (eccentricity = 0.735 ± 0.176). This allows a subsequent analysis of wound healing of circular excision wounds as promoted by each respective therapy, as emerging differences would likely not be a result of wound geometry.

**Figure 3:**
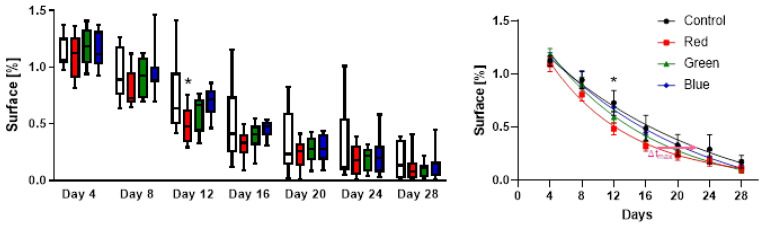
Effects of pulsing LED photobiomodulation therapy of different wavelengths on wound size in percentage changes of wound area relative to day 0 in an excision wound model in diabetic (db/db) mice. The plot on the left shows the median and distribution and the plot on the right shows the mean ± SEM including exponential decay regression fits. Wounds were excised and treated with PBM for 6 min every other day for 28 days. Starting from day 8 after surgery, red and green light showed reduced wound area compared to the control with a maximum delay of 5.1 days over the red group past day 16. *n* = 8, * *p* < 0.05 compared to the not illuminated control group.

Basic wound healing progression was analyzed by comparison of the area of non-enclosed wound surface (Figure 3, left). The average wound area increased during the first 4-day interval in all groups and started to decrease thereafter. At day 8, a trend of a reduction in wound size compared to the control was observable in the red and green light groups, but not in the blue light group. The difference in average relative wound area compared to the control was significant (F(3194) = 4.37, *p* = 0.0253) on day 12 in the red light-treated group (48.6 ± 16.5%) compared to the control group (73.1 ± 33.4%). A visible, yet not significant trend was observed in the green light group (59.6 ± 16.2%). In contrast, there was only a marginal, non-significant reduction compared to the control in the blue light group (69.2 ± 13.7%) compared to the control. These trends of beneficial red and green light were maintained during the rest of the observation period through day 28, but were not significantly different from the control as wounds gradually closed in all groups.

The dynamic wound size was further analyzed utilizing a least squares exponential plateau regression fit to the individual wound size measurements (Figure 3, right). The fitting form for the time-dependent relative wound size reduction from the initial $W_{0}$ is taken to be $\%W(t)=(W_{0}-W_{p})*exp(-k*t)+W_{p}$ with plateau $W_{p}$ and rate constant k. The red light treated group achieves a %50 wound size reduction after 12.2 days, while the control group does not reach this milestone until day 16.7. This delay in healing further increases until it reaches a maximum of 5.1 days at 30.7% of the initial wound size at day 17.3. Green and blue light therapies show similar trends, but at shorter delays.

**Figure 4:**
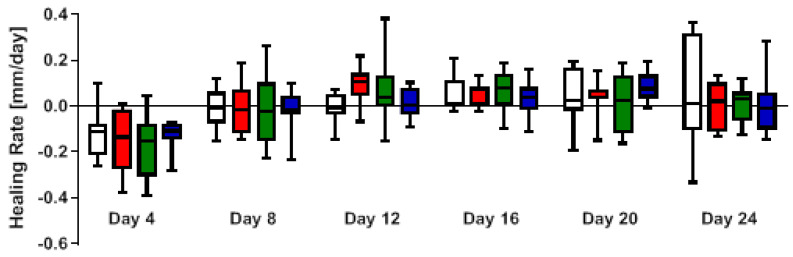
Effects of Repuls pulsing LED light therapy on the wound healing rate in a diabetic wound healing model in mice over a center-weighted 4-day range. Wounds were excised and treated with different wavelengths for 6 min every other day for 28 days. The healing rate was calculated using data from the wound surface. The color of the bars shows the color of PBM wavelength; from right to left control, red, green and blue light, respectively. *n* = 8.

These results coincided with the assessment of wound healing rate, which was analyzed for all interior time points averaged over a center-weighted 4-day window (±2 days in each direction), shown in Figure 4. Due to the increase in wound size, measured on day 4, the mean wound healing rate shows a negative value at this time point. At day 8, the initial wound size was reached again, followed by an early onset of increased wound healing rate at day 12 with red light therapy, with 0.092 ± 0.087 mm/day, and green light therapy, with 0.063 ± 0.155 mm/day. At day 16, similar healing rates were observed in all groups.

**Figure 5:**
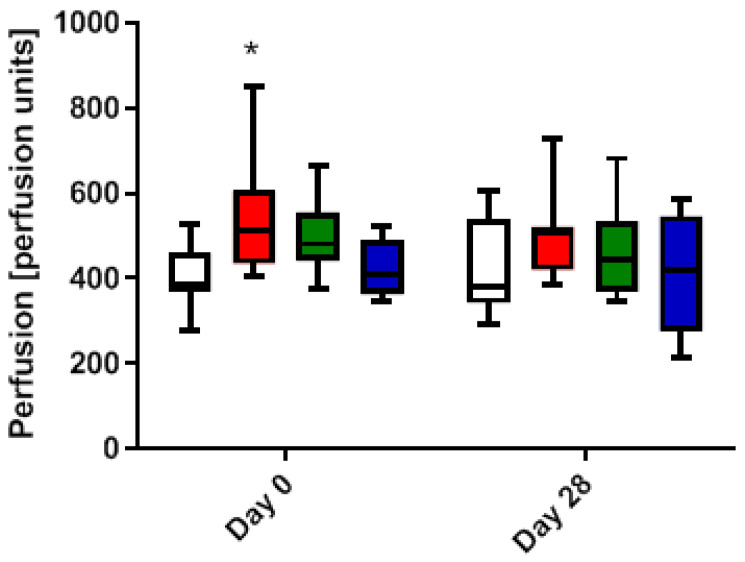
Effects of pulsing LED light therapy by Repuls of different wavelengths on perfusion in the wound given in arbitrary units [AU] immediately after treatment. Wounds were created on day 0 and treated every other day for 6 min for 28 days. Perfusion was analyzed directly after treatment on day 0 and day 28 by laser Doppler imaging (LDI). The color of the bars shows the color of PBM wavelength; from right to left control, red, green and blue light, respectively. *n* = 8, * *p* < 0.05, compared to the not illuminated control.

To analyze the superficial blood flow in the wound area, an LDI (laser Doppler imaging) was performed on day 0 and on day 28 immediately after the illumination. The effect on tissue perfusion showed a wavelength dependency. A significant (F(3,28) = 3.746, *p* = 0.0174) increase in blood flow (Figure 5) after treatment with red (544.1 ± 142.5 AU), and a trend wise increase after treatment with green (495.1 ± 88.5 AU) and blue light (419.6 ± 66.5 AU), was observed on day 0. On day 28, no significant differences in blood flow from red (502.4 ± 105.6 AU), green (459.6 ± 112.7 AU) and blue light (412.7 ± 138.2 AU) were observed, although a similar trend wise increase as on day 0 was persistent in all groups.

**Figure 6:**
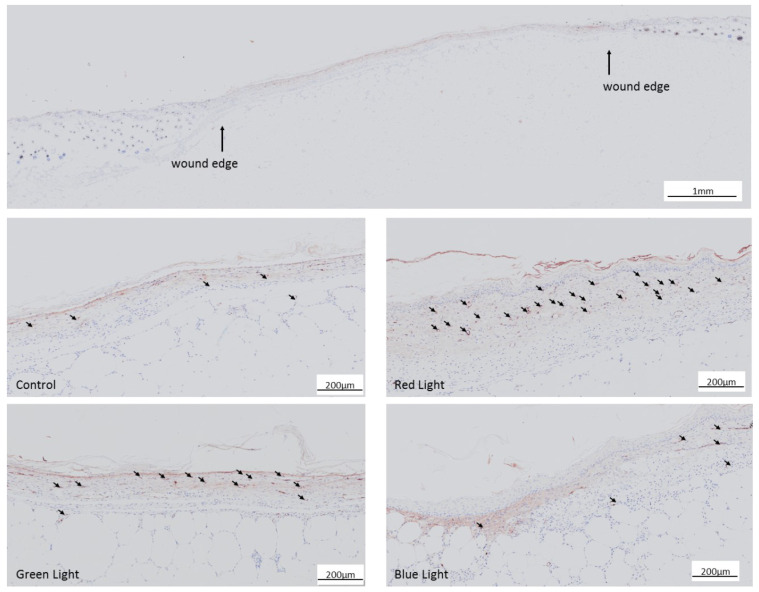
Example images of effects of pulsing light therapy of different wavelengths on the formation of new blood vessels in the wound area after the treatment period of 28 days. Blood vessels were visualized by vWF immunohistochemical staining and counted in the region of interest (ROI). Black arrows indicate vWF-positive blood vessels. The upper large picture shows an overview of the wound. The lower four pictures show blood vessel formation in the not illuminated control group and in the light treated groups. The shown area is within the wounded area. Black arrows point at examples of blood vessels.

**Figure 7:**
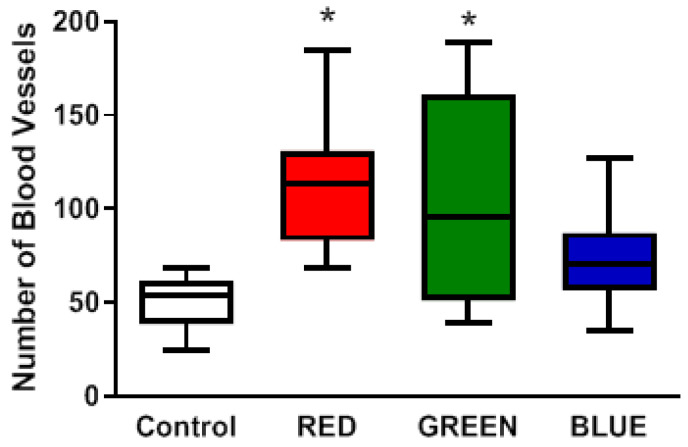
Effects of pulsing LED light therapy on the formation of new blood vessels in the wound area after the treatment period of 28 days. Wounds were excised and treated with different wavelengths for 6 min every second day for 28 days. Blood vessels were visualized by von Willebrand factor (vWF) immunohistochemical staining and counted in the region of interest (ROI), 2 × 2 mm in the middle of the wound surface. * *p* < 0.05 compared to the control group.

In order to analyze blood vessel formation, immunohistochemical staining of vonWillebrand factor (vWF) was carried out on day 28 after euthanasia of the animals. The blood vessels were counted in a 2 × 2 mm area of interest (ROI) in the center of the wound. The number of blood vessels (Figure 6 and Figure 7) was significantly increased (F(3,22) = 3.931, 0.0218) in the wounds treated with red (115 ± 36, *p* = 0.0182) and green light (109 ± 52, *p* = 0.0338). Wounds treated with blue light showed a positive but not significant increase in blood vessel formation (73 ± 27).

**Figure 8:**
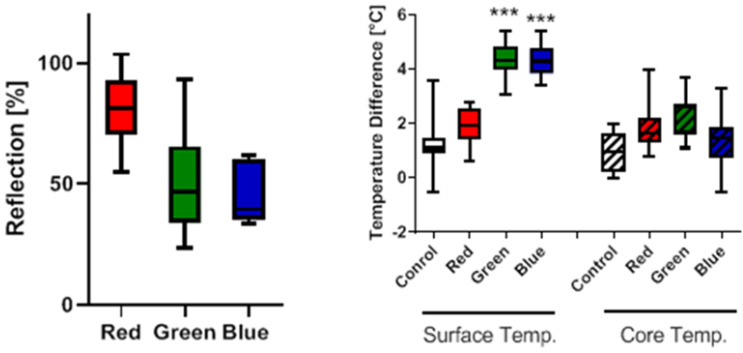
Physical effects of pulsing Repuls LED photobiomodulation therapy of different wavelengths in the treated tissue. Longer wavelengths were reflected to a higher extent (**left**). This effect is associated with a limited rise in wound surface temperature in the red light group (**right**). However, as red light penetrates tissue deeper, red light induced the highest rise in the core body temperature of the animals. *** *p* < 0.001 compared to the control group.

Throughout the treatment, both wound size and wavelength affected the amount of light being absorbed and scattered by the surrounding tissue. In this experiment, the radiative power for each wavelength was nominalized but the absorbed and transmitted energies of the therapy are beyond an experimental evaluation. In lieu, Figure 8 (left) shows the remaining fractional reflection of light intensity of early wounds as an estimate of the power not penetrating the wound or deeper tissue. As expected, red light was mostly reflected, as evident by the surface color, commensally indicating the least energy penetrating the animal. Since the therapy was set calibrated for a constant illumination power, this demonstrates, in turn, that the most energy is being deposited at the wound site for blue light therapy.

The volume, which, together with the energy, is deposited, also varies dependent on the wavelength. Figure 8 (right) shows the significant increase in wound temperature at the end of the treatment. Shorter wavelengths result in larger surface heating, while penetrating longer wavelengths result in a core temperature rise.

**Figure 9:**
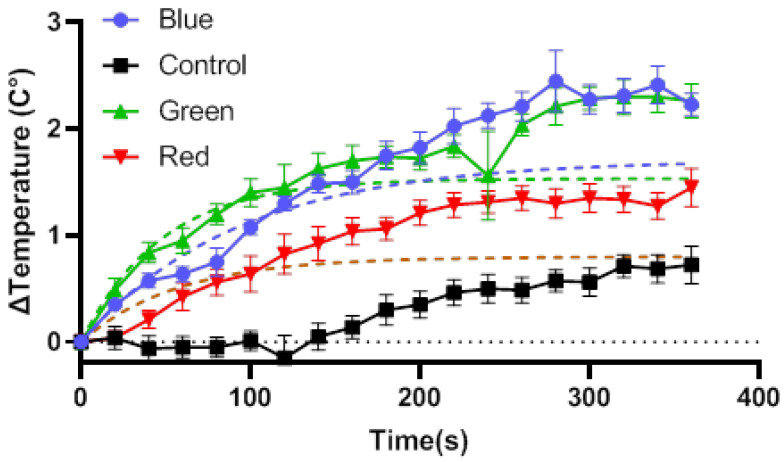
Mean wound surface temperature readings throughout the therapy session taken at 20 s intervals with error bars showing SEM. Dashed lines indicate the exponential plateau regression fit of irradiative warming when colors are corrected for control group kinetic temperature increase.

To obtain better insight into the governing thermal mechanism, further analysis of the wound surface temperature dynamics during therapy was performed. Figure 9 shows the continually increasing mean wound surface temperature when exposed to the light therapy. Noticeably, there is no change in wound surface temperature in the control group for the first 140 s of the sham treatment. Only thereafter has enough time passed for the internally built up thermal energy due to kinetic activity and induced stressors to be transported to the surface.

In order to correct for the light deposition of thermal energy, we need to correct for the inherent internal heating of the control group by treating it as the zero-external heating value. The dashed lines in Figure 8 show that the corresponding control group corrected least squares exponential plateau regression fits to the individual temperature readings. The fitting form $∆T(t)=T_{s}*[1-exp(-k*t)]$, where $T_{s}$ is the long-term thermal steady state equilibrium with rate constant k. For each color therapy at steady state, once net energy transfers have equilibrated, the wound surface heating due to irradiation alone is in the range of 1.575 to 1.877 °C for blue, 1.436 to 1.638 °C for green, and 0.7220 to 0.8869 °C for red, given a 95% confidence interval.

**Figure 10:**
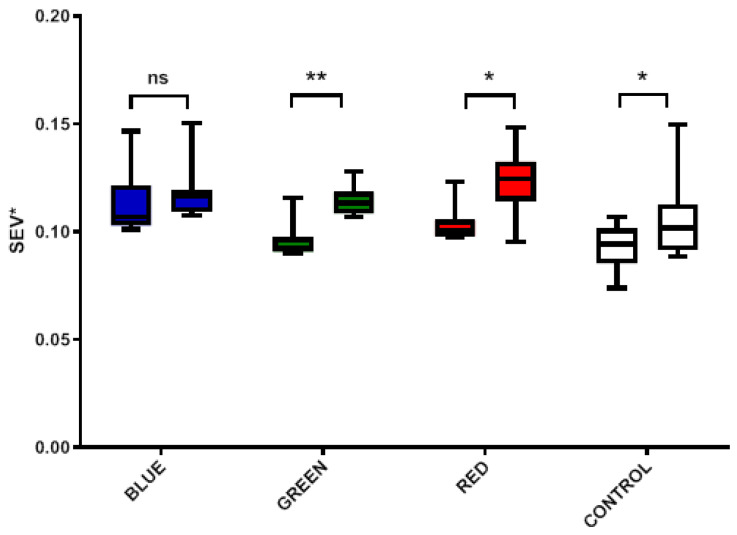
Mean standardized erythema value (SEV*) pre/post comparison per treatment group showing statistically significant increases in all groups except for blue. ns non-significant, * *p* < 0.05, ** *p* < 0.01 compared to the control group.

Figure 10 shows groupwise comparisons of the mean standardized erythema value (SEV *) pre/post comparison, which was obtained by the Scarletred^®^Vision system. The image analysis data shows an increase for all colors over the duration of the therapy session. The wavelength dependent variations in observed superficial skin erythema can be seen as the result of dilatation of the blood capillaries. Wilcoxon matched pairs signed rank tests show the statistically significant largest increases commensurate with higher internal body temperatures for red (*p* = 0.0156) and green (*p* = 0.0078), and only a slight change for the control group (*p* = 0.0156). Only the blue group shows no change (*p* = 0.0781) in the SEV *, where large wound surface temperature increases are observed but comparatively little changes in body temperature.

## 3. Discussion

Promising effects of photobiomodulation (PBM) have already been reported in numerous studies. In the present study we show that positive effects of PBM by pulsed LED light on wound healing and vascularization in a wavelength-depended manner. The irradiance at the target was 50 mW/cm^2^, which gives a radiant exposure of 14.4 J/cm^2^ for 6 min therapy in all treatment groups. As expected from previous studies, red light showed significant effects compared to untreated controls, while green light was also effective. The limited effects of blue light might be associated with light-dependent generation of reactive oxygen species (ROS) [17], which were reported to be predominantly produced at energy densities above 7 J/cm^2^, with blue light inducing the production of damaging concentrations of ROS of mitochondrial origin [18].

Studies using red laser light, such as Kaviani et al., reported that light of 685 nm significantly decreased the size of DFUs in patients compared to a placebo control group [19]. Significantly increased wound contraction was observed by Kajagar et al. in a clinical study investigating the effects of light with 660 nm combined with 850 nm wavelength [20]. Feitosa et al. used a laser with 632.8 nm to decrease the wound size of DFUs in patients and reported significant successes [19]. Additionally, the meta-analysis of randomized controlled trials [21], including the studies of Kaviani et al. and Kajagar et al., showed a significantly enhanced healing rate, diminished ulcer area, as well as a reduced recovery time of DFU patients in comparison to the control groups after low-level laser therapy (LLLT) treatment (wavelength range: 400–904 nm). Publication bias risk analysis demonstrated a low risk with a sensitivity, indicating that the results have strong reliability [21].

Most of the reported studies in the past were performed with laser light. Due to its advantages, including easier handling and lower costs, non-coherent LED light represents an alternative technology. Therefore, recently, more and more treatment options are being performed based on LED light, which was also the case in the present study. Comparative studies by Nishioka et al. and Agnol et al. demonstrated that therapies with laser and LED have similar effects on tissue regeneration and angiogenesis in vivo [22,23].

The previously mentioned studies, as well as most of the studies found in the literature investigating the effects on wound healing in diabetic foot ulcers used light within the red and infrared light spectrum, which currently holds the status of the gold standard in PBM [12].

Delayed wound healing affects wound sizes and its closure. Therefore, the main parameter investigated in this study was wound surface, wound healing rate, vascularization, as well as the overall wound geometry. As seen in Figure 2, the wounds gape initially, a typical behavior of wounds prior to the healing process [24]. After day 4, wound areas in all groups start to decrease consistently, with red and green light treated wounds showing a distinct trend of accelerated closure compared to the control group. This positive trend by red and green light was observable throughout the healing process and reached significance at day 12 in the red light treated group. Comparable stimulating effects of red (700 nm) and green (530 nm) LED light were also observed by de Sousa et al. in an in vitro proliferation assay of rat fibroblasts [25]. Positive effects of green light were also reported by Fushimi et al. using green LED light with 518 nm in both a wound healing model in mice and an in vitro model investigating mRNA and protein levels of cytokines secreted by human fibroblasts during wound healing [26]. In the present study, blue light with the same radiant exposure of 14.4 J/cm^2^ was ineffective, which stands in contrast to the study of Adamskaya et al., where the examined blue light in an excision wound model in rats resulted in significantly decreased wound size [27].

The accelerated wound healing of red and green light treated wounds in our study is also reflected by the wound healing rate (Figure 3), which was significantly increased at day 12 in the red and green light groups. Significantly increased wound healing rates were also observed in studies investigating red and infrared laser light in in vivo studies in rats and clinical studies [28,29,30].

Functionally, vascularization and the enabled reperfusion is a pivotal factor in wound healing and essential to ensure proper blood circulation and tissue integrity [31]. To include these aspects of wound healing, vascularization status was determined by assessing blood vessel presence in the regenerated wounded area. All tested wavelengths increased blood vessel formation, with the strongest effect found for red and green light. Similar angiogenic effects were previously reported by Cury et al. in a skin flap rat model using 660 nm and 780 nm lasers [32]. Zaidi et al. described increased blood vessel formation after red light treatment in an ischemic hindlimb mouse model [33]. We have shown before that angiogenesis and tissue perfusion in a rodent flap model can be significantly enhanced by red light, confirming our observations in the present study [34]. In the cited paper, blue light also showed a beneficial effect, which was not detected in the present study. This could be due to the fact that in the previously performed study ischemia-disturbed wounds were investigated, which added another possible layer of interaction of underlaying mechanisms. Under these conditions, nitrite is activated as an internal pool for nitric oxide (NO), high levels of NO are induced, and this molecule can be best targeted with blue light [14]. In accordance with our data, the stimulating effects of not only red but also green LED light were also confirmed in different in vitro studies using endothelial cells [35], as well as cells of the stromal vascular fraction [36].

We also demonstrated in this study that photobiomodulation led to increased blood perfusion, which was clearly shown by laser doppler imaging (LDI). Significant differences were detected at day 0 in the red and green light group, but not in the blue light group. On day 28, this pattern could also be detected; however, the trends did not reach statistical difference and were not associated with the significantly increased blood vessel counts in the red and green light group. This discrepancy might be explained by the fact that the performed LDI analyses were too insensitive and were only able to detect larger differences in perfusion. The significant increases at day 0 can obviously not be connected to angiogenesis, but rather to temperature increases caused by light, despite the fact that photobiomodulation is also termed cold light, which was also used in the present study to prevent temperature effects. To analyze the physical effects, reflection data in the wounds, as proxy data for tissue absorption, as well as both surface wound temperature and core body temperature of the treated mice, were recorded. Once again, a shift in these parameters was observed in a wavelength-dependent manner. The longer the wavelength was, the more light was reflected at the wound surface with significant differences of green and blue light compared to red light. Inversely, this shows the higher total deposition of energies at the shorter wavelengths. As far as the location of deposition is concerned, the higher absorbance of light of shorter wavelengths led to a significant increase in wound surface temperature after green and blue light treatment. Here, however, we observe a compounded, wavelength-dependent effect of reflection and absorption. Only a comparable small fraction of the higher red wavelength’s energy is being absorbed, as most of it is reflected and the absorption is spread out over a larger volume due to the long wavelength’s increased penetration depth. In contrast, the deposited energy increases for the shorter wavelengths and, in addition, primarily accumulates at the wound site due to decreasing transmissivity.

For this type of small-rodent model, the deposited light energy per animal mass is not insignificant. For one, we observe an internal temperature increase in the control group during sham treatment. This can likely be ascribed to an increased stress level and the confined space allocated during treatment, which better retains body heat. Wound surface temperature readings indicate the same change. For two, irradiative energy deposition with the animal contributes to additional warming. Penetrating longer wavelengths results in a larger increase in core temperature due to the increased deposition depth beyond the wound, even though the animal received a smaller amount of energy. Convective heat transfers are effectively mediated by the animal’s vascularly system, resulting in a homogeneous temperature increase in the body. In contrast, the shorter, more energetic wavelengths lead to higher localized surface wound heating, while only resulting in moderate core temperature rises. Here, cooling occurs due to less effective radiative cooling at the surface, while convective processes are diminished due to low local perfusion volumes. This effect is quite evident and should be considered as an important parameter in controlled small animal studies. As an increased wound temperature can correlate with decreased wound healing and wound bed score [37], the choice of the wavelength used has to be carefully considered.

In conclusion, we demonstrated that PBM by both pulsed red and green LED light has the potential to accelerate wound closure and increase angiogenesis of excision wounds in diabetic (db/db) mice. It remains to be elucidated whether or not the combination of two or more effective wavelengths leads to synergistical effects. PBM provides an effective, non-invasive and comfortable treatment for chronic wounds, such as diabetic foot ulcers. In order to offer the most effective treatment to patients, energy densities, duration and periods of PBM and potential combinations of red and blue or green light have to be compared in the future.

## 4. Materials and Methods

### 4.1. Animal Model

C57BL/KsJm/Leptdb (db/db) mice were obtained from Charles River and Janvier Labs, housed three per cage in a 12-h light/dark cycle, and provided standard laboratory food and water ad libitum. After an acclimatization period of 14 days, the below-mentioned surgical procedure (dorsal excision) was performed. The observation period after surgery was 28 days.

Thirteen-week-old genetically diabetic db/db mice were used for the experiment. The animals were weighed, anaesthetized with 3% isoflurane (Abbott GmbH., Vienna, Austria) and had their blood glucose levels checked via an Akku-Chek Go glucose meter (Roche Diagnostics, Mannheim, Germany). All animals exhibited severely increased blood glucose levels above ≥300 mg/dL. Eight mice were used in each group. The animals’ dorsal hair was shaved and completely removed by using a depilatory cream. The skin was cleaned with alcohol and a ø 1.4 cm full-thickness skin wound was excised under aseptic conditions on the mid-dorsum. Subsequently, 200 μL of transparent hydrogel matrix (NU-GEL, KCI Medizinprodukte GmbH, Vienna, Austria) was topically administered. Then, the wound was covered with transparent Suprasorb F (Lohmann & Rauscher, Schoenau, Austria) to provide a standardized moist wound environment. The animals received an adequate pain management of 0.05 mg/kg Buprenorphine s.c. (Buprenorphin, Richter Pharma, Wels, Austria) 2× daily at intervals of 6–12 h post-surgery for up to 2 days after intervention, as well as 0.15 mg/kg Meloxicam (Meloxicam, Boehringer, Vienna, Austria) daily for the first four days. Post-operative treatment comprised of warming the animals until end of anesthesia and a subcutaneous Ringer lactate infusion depot. Treatments and analyses were performed as stated below. On day 28, the animals were sacrificed in deep isoflurane anesthesia by intracardiac injection, whereupon the wound area was harvested for histologic analysis.

### 4.2. Photobiomodulation Therapy

The first therapeutic application (day 0), according to group allocation, was performed. Application of light therapy was repeated every second day without anesthesia in a custom made container. Dressing changes were performed every four days, during which fresh hydrogel was applied.

**Figure 11:**
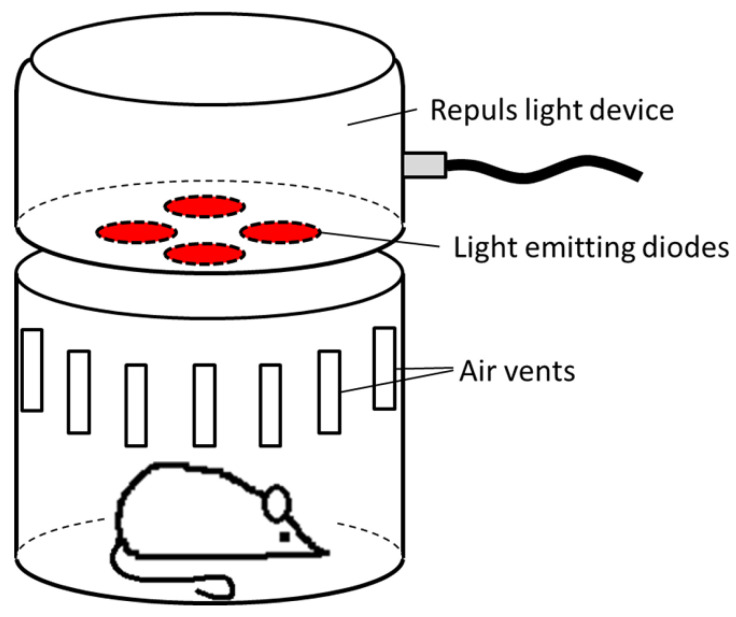
Scheme of PBM treatment of db/db mice in a container fitting the diameter of the LED device by Repuls.

PBM was performed with red (629 nm), green (540 nm) and blue (470 nm) light. Irradiation time was 6 min (through the transparent wound cover membrane), and all therapy LEDs used a pulse frequency of 2.5 Hz, a duty cycle of 50%, and were normalized to 40 mW/cm^2^. These parameters were chosen based on the positive results of previous in vitro and in vivo studies [34,35,36]. The mouse was placed in a transparent, cylindrical enclosure of a diameter commensurate with the size of light source with surrounding ventilation slots for cooling, to ensure the animal remains within consistent light exposure throughout the therapy while maintaining some mobility (Figure 11). The vertical source distance to the mouse’s dorsum was kept at 10 cm. The overall dose of each treatment was 14.4 J/cm^2^.

### 4.3. Wound Assessment

Wound size was assessed every fourth day from day 0 through to day 28 during wound dressing changes, using a stereoscopic lens (LifeViz micro, Quantificare, Biot, France) and a Canon Rebel XSi camera (Canon, Ota City, Tokyo, Japan) for 3D wound measurement (circumference, surface, average depth). Stereoscopic wound analysis parameters were evaluated using DermaPix Pro 2.28.5. Skin erythema was captured by using a smartphone (iPhone 6 Plus, Apple Inc., Cupertino, CA, USA) with installed CE medical device software Scarletred^®^ Vision plus a Scarletred^®^ Skin patch, which resulted in auto white balanced and color corrected skin images over treatment time. The images were consecutively uploaded and analyzed within the Scarletred^®^ web platform (SCARLETRED Holding GmbH, Vienna, Austria) by using the standard erythema value (SEV *) algorithm [38,39,40].

Additionally, the animals were scanned via laser Doppler imaging (Moor, UK) under isoflurane inhalation anesthesia on the day of surgery (day 0) and on day 28 to assess superficial tissue perfusion. Results for the post-operative and post treatment scans were calculated in percentage of arbitrary units (AU) from baseline, pre-operative scans.

The wound healing rate for every 8th day was calculated using the data from the wound surface (A) and perimeter (P) evaluated at time points T1 and T2.

(1) $$\mathrm{healing}\;\mathrm{rate}\;\mathrm{in}\;\mathrm{mm}/\mathrm{day}\;=\;\frac{\frac{A1-A2}{\left(P1+P2\right)*0.5}}{T2-T1}$$

### 4.4. Histology and Immunohistochemistry

For histological evaluation, haematoxylin, eosin and immunohistochemical vWF stainings were performed according to standard protocol. Briefly, formalin-fixed paraffin-embedded tissue specimens were cut in 5 μm thick sections and de-paraffinized. Immunohistochemical stainings were performed in a Lab Vision Autostainer 360 (Thermo Scientific, Waltham, MA, USA). Anti-vWF (1:100, M-20, sc-1506-G, Santa Cruz Biotechnology, Santa Cruz, CA, USA) was used as the primary antibody.

### 4.5. Quantification of Stainings

The vWF-stained whole sections were scanned with an Olympus BX61VS scanning microscope (Olympus Austria GmbH, Vienna, Austria) at ×20 magnification. The region of interest was defined to be 2 × 2 mm in the center of the regenerated wound tissue.

### 4.6. Temperature and Reflection Measurements

At given timepoints, the effects of light treatment on both wound surface temperature and body core temperature were recorded. Wound surface temperature readings were obtained both at the start and immediately after the conclusion of individual therapy sessions via a contactless infrared thermometer PhotoTemp MX (Raytek, Bremgarten, Switzerland). Body temperature readings were concurrently obtained via a rectal probe using a Fluke 52 Series II thermometer (Fluke Austria GmbH, Brunn, Austria).

Stereoscopic wound images were taken flanked by a grey/white reference card of 18% and 90% calibrated reflectance, respectively (Kodak, Rochester, New York, NY, USA), under monochromatic therapy illumination only. The sRGB color space recorded images were converted to a purely gamma-based color space Adobe RGB 1998 (ICC profile by Adobe Systems Incorporated, San Jose, CA, USA) and subsequently linearized. Average wound reflection brightness was obtained by the stereoscopic average of their intensities as a fraction of the reference card measurement. All image analysis was performed using Mathematica 12 (Wolfram Research, Champaign, IL, USA).

### 4.7. Statistical Methods

Data obtained were statistically evaluated using GraphPad Prism 7 software (GraphPad Software Inc., LA Jolla, CA, USA). Throughout, all group comparisons were done using a two-way ANOVA to analyze the effect of individual light colors and data passed the Shapiro–Wilk test of normality. Post hoc multiple group comparisons utilized Dunnett corrections. Data are expressed as mean ± standard deviation. All tests were performed in a two-sided manner at a significance level of α = 0.05, and *p*-values equal or below 0.05 were considered as statistically significant. Figures showing box-and-whisker plots indicate central quartiles with min/max whiskers, and summary plots show mean values ± SEM.

## Data Availability

All data and analysis are available on request from the corresponding author.
